# Systematic Approach for Drug Repositioning of Anti-Epileptic Drugs

**DOI:** 10.3390/diagnostics9040208

**Published:** 2019-11-30

**Authors:** Younhee Ko, Chulho Lee, Youngmok Lee, Jin-Sung Lee

**Affiliations:** 1 Division of Biomedical Engineering, Hankuk University of Foreign Studies, Kyoungki-do 17035, Korea; 2 Department of Clinical Genetics, Department of Pediatrics, Yonsei University, College of Medicine, Seoul 03722, Korea; 3 Department of Pediatrics, Yonsei University, College of Medicine, Seoul 03722, Korea

**Keywords:** epilepsy, drug-target network, drug repositioning, precise medicine, NGS

## Abstract

Epilepsy is a central neurological disorder affecting individuals of all ages and causing unpredictable seizures. In spite of the improved efficacy of new antiepileptic drugs and novel therapy, there are still approximately 20%~30% of patients, who have either intractable or uncontrolled seizures. The epilepsy drug–target network (EDT) is constructed and successfully demonstrates the characteristics and efficacy of popularly used AEDs through the identification of causative genes for 60 epilepsy patients. We discovered that the causative genes of most intractable patients were not the targets of existing AEDs, as well as being very far from the etiological mechanisms of existing AEDs in the functional networks. We show that the existence of new drugs that target the causative genes of intractable epilepsy patients, which will be potential candidates for refractory epilepsy patients. Our systematic approach demonstrates a new possibility for drug repositioning through the combination of the drug-target and functional networks.

## 1. Introduction

Epilepsy is the most common neurological disorder with heterogeneous causes, affecting 1%~2% of the population. The majority of patients with epilepsy can be effectively treated, given an accurate diagnosis and appropriate medication. In order to select efficientdrug regimens, accurate diagnosis for the types of epilepsy is very critical [1,2], and the goal of epilepsy management is complete control of seizures with little or no adverse effects from the appropriate antiepileptic drug (AED). However, most of the existing therapies for epilepsy patients have focused on symptomatic treatment with such drugs instead of identifying the leading cause of epilepsy. There are general sorted antiepileptic drug lines of treatment for specific types of epilepsy [3,4]. If a single drug cannot control the seizure, then a combination of several AED [5,6,7] is provided, or a ketogenic diet is recommended [8,9,10,11,12]. In spite of the improved efficacy of new AED drugs and novel therapies, there still approximately 20%~30% of patients who have either intractable or uncontrolled seizures [13,14].

A recent improvement in the cost and accuracy of whole-exome sequencing (WES) has enabled the accurate diagnosis of genetic diseases and the identification of the causative variants of epilepsy patients. Especially for patients with refractory epilepsy, WES is popularly used to identify accurate causative genes through the trio analyses, which would be very helpful in providing an accurate diagnosis of epilepsy patients with unknown origin. The understanding of biological mechanisms of existing AEDs and pathologies of epilepsies can lead to a dramatic advance of AED development. Thus, it is a feasible strategy to predict appropriate drugs based on the causative genes or perturbed biological pathways of refractory epilepsy patients, which shows the great impact of precise medicine [15,16,17,18,19]. 

In this study, we construct the epilepsy drug–target network (EDT) and successfully demonstrate the characteristics and effectiveness of popularly used AEDs and the pathological mechanisms of existing AEDs. Especially, we discovered that the causative genes of most intractable patients were not the targets of existing AEDs, as well as that they are located far from the etiological mechanisms of existing AEDs in the functional networks. Finally, we show the existence of new potential drugs, which target the causative genes of intractable epilepsy patients, which will be a new candidate for refractory epilepsy patients. Our systematic approach demonstrates a new possibility for drug repositioning through the combination of the drug-target and functional networks.

## 2. Materials and Methods

### 2.1. Clinical Exome Sequencing and Data Analysis

We utilized the TruSight One sequencing panel providing comprehensive coverage of <4800 clinically known disease-associated genes. Sequencing was performed using the Illumina MiSeq. The sequenced reads were mapped to the human reference (UCSC hg19) with the Burrows-Wheeler aligner (BWA), and variants were identified with the genome analysis toolkit (GATK). Sequence variants were filtered according to various quality parameters. The segregation of the pathogenic mutation in the family was verified by automatic Sanger sequencing using the BigDye (Applied Biosystems, v3.1, Foster City, CA, USA) protocol on a 3130 XL analyzer (Applied Biosystems). 

### 2.2. AED Lists

In this study, we focused on 13 popularly used AEDs covering classical AEDs as well as new AEDs: carbamazepine, phenytoin, valproic acid, ethosuximide, primidone, phenobarbital, lamotrigine, oxcarbazepine, topiramate, zonisamide, gabapentin, vigabatrin, and levetricacetam. Note that benzodiazepine and tigabine were excluded due to missing drug-target information in the DGI Database. 

### 2.3. Disease Causative Genes

GeneCards is a comprehensive database providing information on all annotated and predicted human genes by integrating genomic, transcriptomic, proteomic, genetic, clinical, and functional information. We downloaded 2208 epilepsy-associated genes from GeneCards. After filtering out the genes without EntrezID mapping information, 2116 epilepsy-associated genes are used in this study.

### 2.4. Drug-Target Interactions (DGIs)

The Drug-Gene Interactions Database (DGIdb, www.dgidb.org) is a resource describing drug–gene interactions and gene druggability. DGIdb integrated 27 sources describing drug–gene interactions and druggable gene categories including DrugBank or the Therapeutic Target Database (TTD). We complied all drugs targeting any of the 2116 epilepsy-causative genes, which led to 2480 drugs. Some of the ambiguous drug names are filtered out. Among 2116 epilepsy-causative genes, 694 genes are targeted by current drugs in DGIdb.

### 2.5. Network Database

The STRING 9.1 network database, one of the largest databases of direct protein–protein interactions and indirect functional interactions constructed from various data sources, was used. It contained approximately 20,772 proteins, with 2,425,315 interactions among them. We extracted the interactions among 2116 epilepsy-causative genes from the STRING protein-protein interaction (PPI) network, which covers 4815 interactions among them and then filtered out the low-quality interactions. Entrez gene IDs were used to map disease-associated genes to the corresponding proteins and drug targets.

### 2.6. Construction of EDT-DRUG Network

**Figure 1:**
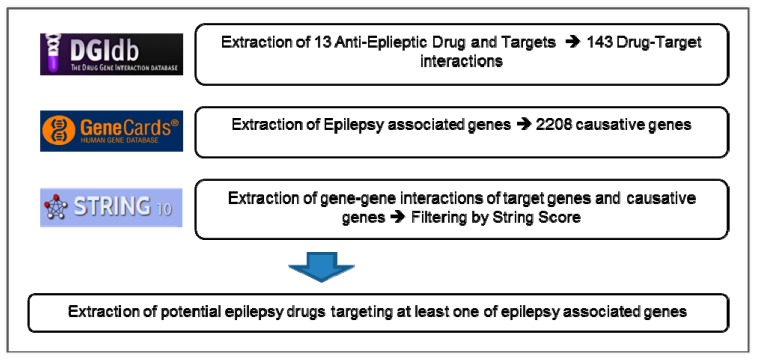
Data Integration Schema: Data source for drug-target information, epilepsy causative genes, and protein-protein interaction and their statistics.

We constructed a drug-target network covering 2116 epilepsy-causative genes and 13 clinically used epilepsy drugs and 636 putative drugs targeting any of epilepsy-causative genes and their interactions (Figure 1). The constructed network was visualized by Cytoscape v3.4.0.

## 3. Results

### 3.1. Characteristics of Clinically Used Anti-Epileptic Drugs

We collected 13 clinically used anti-epileptic drugs (Table 1) and 2116 genes known to be associated with epilepsy from the GeneCard database. We integrated the drug-target interaction information from the DGI database and protein-protein interactions (PPIs) from String databases, which leads the total of 143 drug-target interactions and 4815 PPIs. 

**Figure 2:**
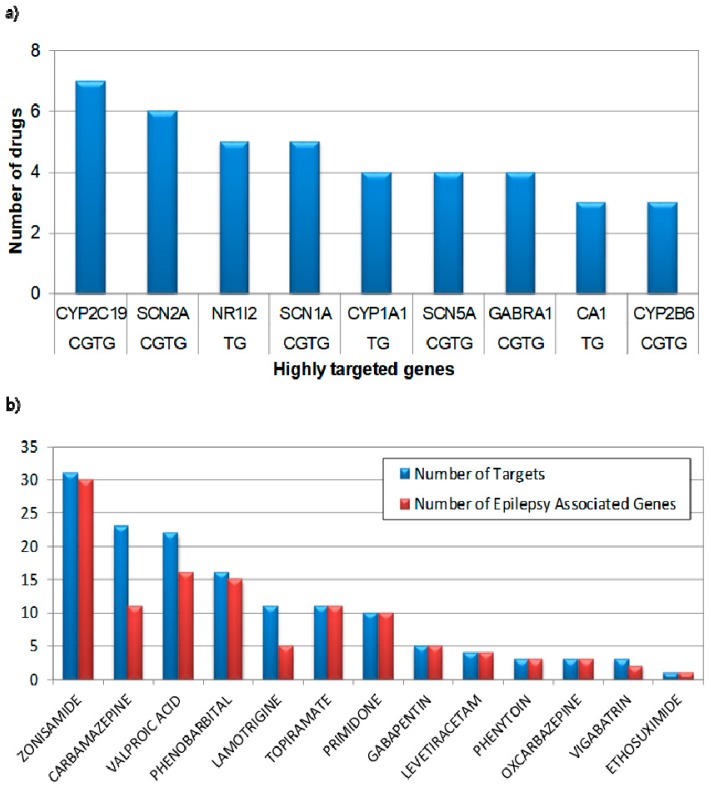
Clinically used anti-epileptic drugs (AED), Targets, and epilepsy-associated genes. (**a**)Highly targeted gene by AEDs, the *x*-axis represents the target genes of 13 clinically used AEDs, the *y*-axis represents the number of drugs targeting the genes. CGTG represents that it is an epilepsy-causative gene as well as a target gene of AEDs, and TG represents that it is only a target gene of AEDs (**b**) Target distribution of 13 clinically used AEDs, blue bar represents the number of target genes, red bar represents the number of epilepsy-associated target genes.

First of all, we focused on highly targeted genes by known anti-epileptic drugs and targeted gene statistics of current AEDs. As shown in Figure 2, voltage-gated sodium channel-related genes (e.g., SCN2A, SCN1A, SCN5A) are remarkably ranked as the highly targeted genes, where approximately half of clinically used AEDs are designed to target this specific molecular mechanism. Interestingly, the voltage-gated sodium channels are known to initiate action potentials in brain neurons and are primary therapeutic targets of AEDs and other neurological disorders. Thus, mutations of genes in sodium channels are very critical for genetic epilepsy syndromes. Many AEDs are mostly designed to control this pathological mechanism, which explains the major causes of epilepsy. Our analysis clearly discovered the fundamental importance of voltage-gated sodium channels in epilepsy. In addition, CYP2C19 and NR1I2 (i.e., PXR) [20] are also identified as highly targeted genes by clinically used AEDs. CYP2C19 is a known liver enzyme that acts on 10%~15% of drugs in current clinical uses. Genetic polymorphism of cytochrome P450 genes, such as CYP2C9 and CYP2C19, is especially known for drug resistance in epilepsy patients and it may be the reason that such genes are highly targeted by current AEDs. In addition, the pregnane X receptor (PXR) also plays critical and diverse roles in mediating xenobiotic induction of drug-metabolizing enzymes and transporters. Our results effectively demonstrate that such important AED target genes are strongly associated with pathological mechanisms of epilepsy. To further investigate the characteristic of current AEDs, we showed the number of targets of current AEDs and their known associations with epilepsy. As shown in Figure 2b, traditional AEDs such as zonisamide (26)., carbamazepine, and valproic acid are targeting a relatively large number of genes, which may lead to the large therapeutic effects but unexpected side effects due to the broad targeting of genes. Meanwhile, recently developed drugs such as oxcarbazepine, vigabartrin, and ethosuximide are specially designed to target a relatively small number of genes, which reduce the side effects and drug interactions, although their therapeutic effects are relatively less than traditional ones. Clinically, traditional AEDs reveal more severe side effects than relatively newly developed drugs, which is explained in Figure 2b. In addition, carbamazepine or lamotrigine reveal that they are also targeting many genes that do not have a known association with epilepsy. One of the reasons for this difference is that known associations with epilepsy and its causative genes are not sufficient, or those drugs might play multiple roles for other diseases as well.

**Figure 3:**
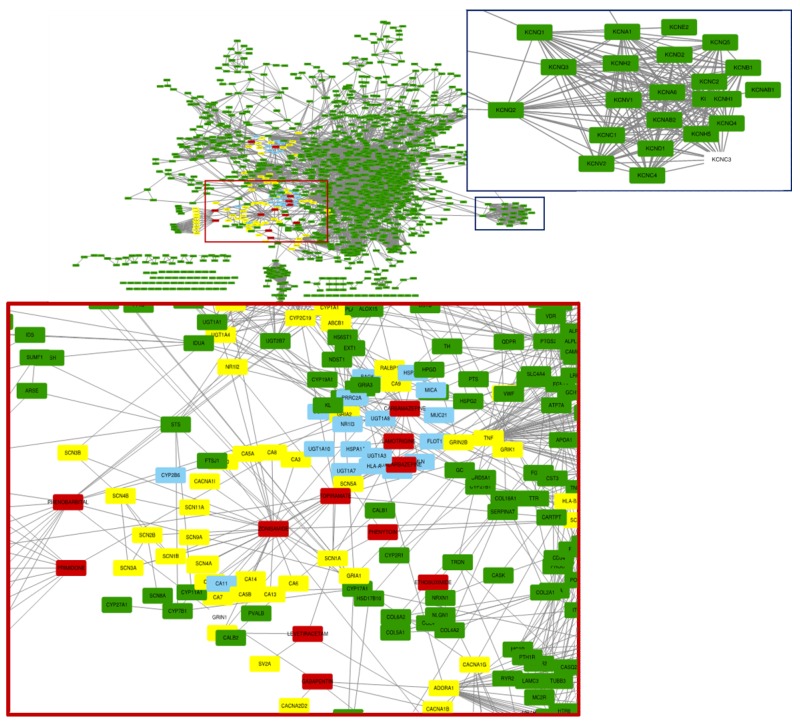
The EDT-drug network; AED-target genes-epilepsy associated gene network, red nodes represent the 13 clinically used AEDs, yellow nodes represent targets of AEDs as well as epilepsy-associated genes, skyblue nodes represent the targets of AEDs, and green nodes represent the epilepsy-associated genes not targeted by current AEDs.

To investigate the relationship between current AED targets and epilepsy-associated genes, we constructed a drug-target-causative gene network covering AED drug-target interactions and protein-protein interactions between epilepsy-associated proteins (Figure 3). Interestingly, as we can see in Figure 3, among a large number of epilepsy causative genes, only a small portion of causative genes are targeted by current used AEDs. Although most of epilepsy causative genes are not targeted by current known AEDs, as shown with green nodes in Figure 3, current drugs show fairly enough therapeutic efficacy to control most of the epilepsy patients. It can be explained by the fact that most of the epilepsy patients share similar etiological factors and current AEDs could well control such pathological mechanisms without the direct targeting of the specific genes. Thus, most of the epilepsy patients under control by current AEDs have pathological problems that are effectively controlled by current AEDs (i.e., yellow nodes in Figure 3), and it reveals evidence for the broad efficacy of current AEDs. However, in the case of intractable epilepsy patients, causative genes neither have direct interactions with the targets of current AEDs nor do they show close proximity with the current targets of AEDs in the PPI network. Thus, the pathological mechanisms of intractable epilepsy patients cannot be controlled by the pharmacological mechanisms of current AEDs.

### 3.2. Epilepsy Associated Genes and Active Targeting Drug

For 2116 epilepsy causative genes, we extracted all known drugs targeting any of such epilepsy associated genes, which lead to the 5657 drug–target interactions from DGI database after filtering. Based on the drugs in DGI databases, 1445 known epilepsy causative genes could not be any of known drug targets and only 671 genes are currently targeted by at least one of existing drugs, leading to a total of 2480 drugs and 5657 drug-arget interactions. Interestingly, there are many well-known drugs targeting epilepsy-associated genes although they are not designed for epilepsy. It shows the new potential of these drugs for drug-repositioning to epilepsy. Since these drugs have already been approved for use in humans and we know that they are actually targeting epilepsy causative genes, they could be very successful candidate drugs for epilepsy patients. Many different drugs with shared targets could control the same or similar molecular pathways and a drug repositioning strategy based on target gene proximity is very promising in suggesting new potential drugs especially for intractable epilepsy patients. In order to extract the potential candidate epilepsy drugs, we identified the drugs having more than three epilepsy causative genes as targets, which might be used for further investigation for drug repositioning to epilepsy (supple 1). 

### 3.3. Identification of Causative Genes from Intractable Epilepsy Patients

Based on our observation, we suspect that the causative genes of such intractable epilepsy patients are neither connected with targets of current AEDs, nor located close to them. Indeed, most of the causative genes identified from intractable epilepsy patients are isolated from the target cluster of current AEDs. For example, the genes associated with voltage-gated potassium channels (e.g., KCNQ1, KCNQ2, KCNQ3, KCNV1, KCNV2, KCNA1, KCND1, KCNG2) are identified as ones of popular causative genes from intractable epilepsy patients, as shown in Figure 3. Thus, recently, a burgeoning number of AEDs has been developed and reported to target voltage-gated potassium channels. It might be the reason that current available AEDs fails to control for epilepsy patients having problems in potassium voltage-gated channels.

### 3.4. Accurate Diagnosis for Epilepsy Patients Through NGS

We observed 60 epilepsy patients who were referred and consented for clinical exome sequencing at the clinical genetics laboratory of Severance Children’s Hospital. Most epilepsy patients were effectively controlled by current AEDs. For some of the intractable epilepsy patients (i.e., 16 patients), ketogenic diet or combination therapy was recommended depending on their manifestations. To investigate the efficacy of current AEDs, we checked the medical history of 16 patients from EMR (electronic medical records). With the investigation of the pathogenic mutations of patients and the efficacy of AEDs, interestingly, we found that epilepsy patients having pathogenic mutations in the genes associated with voltage-gated sodium channels (i.e., SCN8A, SCN2A) are well controlled with clinical AEDs without any serious side effects. Note that the voltage-gated channel pathway is one of the most targeted pathways by current AEDs (Figure 2a and Figure 3), and it may be the reason that patients having pathogenic mutations in voltage-gated sodium channels are well controlled with current AEDs. Among these 16 patients with epilepsy, we identified 4 patients who could not be treated with combination therapy and their pathogenic mutations. Our analysis demonstrated that patients having pathogenic mutations in MECP2, CDKL5, PRODH, KCNQ2 are not controlled well with existing AEDs, which lead to the recurrence of epilepsy after therapy with current AEDs (Table 2). We investigated the causative genes of those intractable epilepsy patients (i.e., MECP2, CDKL5, PRODH, KCNG2) in our AED-target genes-epilepsy associated gene network. Indeed, these causative genes are not the direct targets of current AEDs and are also very far from current targets (Figure 3). That explains the inefficacy of the existing drugs for those intractable epilepsy patients. Furthermore, we may recommend the new candidate drugs for these intractable epilepsy patients be based on their PPI interactions in our network.

### 3.5. NGS for Epilepsy Patients

**Figure 4:**
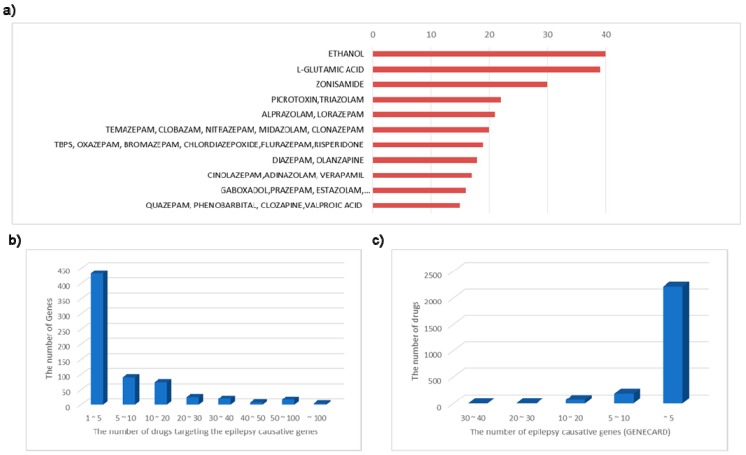
The top ranked drugs targeting epilepsy causative genes and the summary statistics of known drugs and their targets. (**a**) The *x*-axis represents the number of epilepsy causative genes targeted by listed drugs. Note that Ethanol is a top ranked drug targeting 40 epilepsy causative genes. L-Glutamic acid and zonisamide are also top-ranked drugs; each of them targets 39 and 30 genes, respectively. (**b**) The *x*-axis represents the number of drugs targeting the epilepsy causative genes, and the *y*-axis represents the number of genes. Note that most of epilepsy-causative genes (e.g., 433 genes) are targeted by less than five drugs. (**c**) The *x*-axis represents the number of epilepsy-causative genes based on GENECARD and the *y*-axis represents the number of drugs. For example, most of drugs (2209 drugs) target less than five epilepsy-causative genes.

We identified the number of epilepsy causative genes targeted by epilepsy drugs as well as putative drugs. Figure 4a shows the statistics of epilepsy causative genes and where how often they are targeted by currently available drugs. As we can see, among a total of 671 targeted genes, approximately 216 epilepsy causative genes (32.2%) are targeted by only one specific drug. On the other hand, about 22 genes were targeted by at least 50 drugs. Note that 4 among those 21 genes (i.e., HTR2A, CHRM3, HTR1A, HTR2C, and GABRA1) were targeted by over 80 drugs, which shows their high druggability. Interestingly, the 5-hydroxytryptamine receptor family (e.g., HTR2A, HTR1A, HTR2C, HTR1B, HTR7, HTR3A, etc) and the gamma-aminobutyric acid type A receptor family (e.g., GABRA2, GABRA2, GABRA3, GABRA5) are the most targeted genes by clinically available drugs. In addition, HCN channels which can generate hyperpolarization-activated cation currents and be a causative gene for epilepsy, could also be a potential target tailored as an antiepileptic agent [21,22]. The defect in the KCNJ11 gene (Kir2-encoded current) can also cause convulsive disorder. The opener of these channels could be tailored for a specific antiepileptic agent [23,24,25]. Our systematic approach can be effectively applied to identify those causative genes as well as the potential drugs targeting them.

As shown in Figure 4b, most drugs (89%) target less than 5 epilepsy causative genes. Interestingly, those top-ranked drugs report direct or indirect known associations with epilepsy. Zonisamide, picrotoxin, triazolam, alprazolam, and lorazepam, especially, are identified as the drugs targeting at least 20 epilepsy causative genes (Figure 4a). Such broad targeting of drugs may increase effectiveness; however, it may also cause severe unexpected side effects [26]. Therefore, recently developed anti-epileptic drugs reveal a tendency toward targeting a small number of targets.

Also, among top-ranked drugs in Figure 4a, well-known drugs zonisamide and L-glutamic acid [27,28] have critical roles for decreasing the seizures of patients suffering from uncontrolled epilepsy. Meanwhile, most of the top-ranked drugs show the opposite role for epilepsy control by target epilepsy-causative genes. For example, Picrotoxin [29] is used for relieving respiratory distress but plays an important role in establishing GABA as an inhibitory transmitter and can cause seizures by impacting the central nervous system. This side effect is easily predicted from our network, where these drugs access many epilepsy-causative genes and it can cause such unexpected results. Similarly, alprazolam and triazolam are also known to cause seizures occasionally although they are beneficial for patients with major depression. Thus, we need to investigate with caution in interpreting the results. Most of the top-ranked drugs in Figure 4 are identified as drugs causing epilepsy or seizure as side effects, although they have a positive therapeutic effect on another disease. Thus, a target-based drug repositioning strategy should not choose the drugs sharing targets with epilepsy-causative genes, but consider the drug–target interaction types.

## 4. Discussion

In our EDT-drug network, we observed that the voltage-gated potassium channels associated genes are located far from the targets of current AEDs. The voltage-gated potassium channels are known to be one of the commonly perturbed mechanisms in intractable epilepsy patients. Indeed, as shown in Figure 4, we could observe that the current 13 AEDs are not targeting any of the genes associated with this biological process. Recently, there was a newly developed drug, Retigabine [30], which was designed to target voltage-gated potassium channels. Retigabine was the first unique potassium channel opener that worked by activating a part of KCNQ potassium channels in the brain [15] and it was effective for intractable epilepsy patients having trouble in voltage-gated potassium channels. However, there were reported adverse effects including blue skin discoloration and eye abnormalities characterized by pigment changes in the retina [31]. Unfortunately, Retigabine was recently discontinued due to the unexpected adverse effects. Although Retigabine was discontinued, our observation shows a new promise for drug repositioning to recommend novel drugs for intractable epilepsy patients.

In summary, we constructed the epilepsy drug-target network (EDT) and successfully demonstrated the effectiveness of popularly used AEDs and the pathological mechanisms of existing AEDs. Our study contributes to the identification of novel drug repositioning strategies through a combination of the drug–target and functional networks.

## Figures and Tables

**Table 1:** Clinically used AED (anti-epileptic drug)

Anti-Epileptic Drug Name	Type	Usage/Effects	Serious Side Effects
ZONISAMIDE	classical anti-epileptic drug/broad-spectrum AED	used as adjunctive therapy, indicated for partial seizures in adults	Ataxis
CARBAMAZEPINE	classical anti-epileptic drug/narrow-spectrum AED	decreasing nerve impulses that cause seizure and pain, indicated for temporal love epilepsy, grand mal and mixed type seizures	Dermatologic reactions, bone marrow suppression
VALPROIC ACID	classical anti-epileptic drug/broad-spectrum AED	preventing of absence seizures, partial seizures, and generalized seizures	Hepatotoxicity, pancreatitis, congenital malformations
PHENOBARBITAL	classical anti-epileptic drug/narrow-spectrum AED	slows the activity of the brain and nervous system, used for tonic clonic seizure	CNS depression
LAMOTRIGINE	new anti-epileptic drug/broad-spectrum AED	used for focal seizures, tonic-clonic seizures, Lenox–Gastaut syndrome	Stevens–Johnson syndrome
TOPIRAMATE	new anti-epileptic drug/broad-spectrum AED	used for generalized tonic-clonic seizure and developmental delay	Neuropsychiatric reactions, visual field defect
PRIMIDONE	classical anti-epileptic drug	used for grand mal or focal seizure and neuropathic pain	Ataxia, vertigo
GABAPENTIN	new anti-epileptic drug/narrow-spectrum AED	used for partial seizures or neuralgia, affects chemicals and nerves in the body	Hypersensitivity
LEVETIRACETAM	new anti-epileptic drug/broad-spectrum AED	used for partial-onset or tonic-clonic seizures, myoclonic seizures	Psychiatric symptoms, anaphylaxis
PHENYTOIN	classical anti-epileptic drug/narrow-spectrum AED	used for status epilepticus, slow down impulses in the brain causing seizures	Hypotension, arrhythmias
OXCARBAZEPINE	new anti-epileptic drug/narrow-spectrum AED	used for partial seizure, decrease nerve impulses	Hyponatremia, hypersensitivity
VIGABATRIN	new anti-epileptic drug/narrow-spectrum AED	used in combination with other medicines, treats for complex partial seizures	Vision loss
ETHOSUXIMIDE	classical anti-epileptic drug	treat absence seizures	Allergic reactions

**Table 2:** The causative genes of intractable epilepsy patients and representative manifestation.

Patient ID	Symptom	Identified Causative Genes from Whole Exome Sequencing
1	Seizure, Rett Syndrome	*MECP2*
2	Epilepsy, West syndrome	*CDKL5*
3	Early infantile epileptic encephalopathy, West syndrome, mental retardation, microcephaly	*KCNQ2*
4	Epilepsy, metabolic disorder, cerebral palsy, leukodystrophy, mitochondrial myopathy, delayed development	*PRODH*

## Supplementary Materials

The following are available online at https://www.mdpi.com/2075-4418/9/4/208/s1.
