# Supplementary material for: Systematic Approach for Drug Repositioning of Anti-Epileptic Drugs
Source: Diagnostics (Basel). 2019 Nov 30;9(4):208. doi: 10.3390/diagnostics9040208 (PMC6963462; doi:10.3390/diagnostics9040208)
Supplement: Supplementary file 1 [file diagnostics-09-00208-s001.pdf]

ETHANOL	40
L-GLUTAMIC ACID	39
ZONISAMIDE	30
PICROTOXIN	22
TRIAZOLAM	22
ALPRAZOLAM	21
LORAZEPAM	21
TEMAZEPAM	20
CLOBAZAM	20
NITRAZEPAM	20
MIDAZOLAM	20
CLORAZEPATE	20
CLONAZEPAM	20
TBPS	19
OXAZEPAM	19
BROMAZEPAM	19
CHLORDIAZEPOXIDE	19
FLURAZEPAM	19
RISPERIDONE	19
DIAZEPAM	18
OLANZAPINE	18
CINOLAZEPAM	17
ADINAZOLAM	17
VERAPAMIL	17
GABOXADOL	16
PRAZEPAM	16
ESTAZOLAM	16
FLUDIAZEPAM	16
HALAZEPAM	16
CLOTIAZEPAM	16
AMITRIPTYLINE	16
PYRIDOXAL PHOSPHATE	16
QUAZEPAM	15
PHENOBARBITAL	15
CLOZAPINE	15
VALPROIC ACID	15
OCINAPLON	14

SAFINAMIDE	14
NICOTINE	14
PACLITAXEL	14
ARIPIPRAZOLE	14
NIFEDIPINE	14
GLYCINE	13
ELLAGIC ACID	13
DOCETAXEL	13
ADENOSINE TRIPHOSPHATE	13
CHLORPROMAZINE	13
GLUTATHIONE	12
HYDROCHLOROTHIAZIDE	12
LOXAPINE	12
MIRTAZAPINE	12
CARBAMAZEPINE	11
PENTOBARBITAL	11
SECOBARBITAL	11
HEXOBARBITAL	11
TOPIRAMATE	11
2'-MONOPHOSPHOADENOSINE 5'-DIPHOSPHORIBOSE	11
IMIPRAMINE	11
ZIPRASIDONE	11
FELODIPINE	11
FLUFENAMIC ACID	10
AMOXAPINE	10
APROBARBITAL	10
AMOBARBITAL	10
HEPTABARBITAL	10
BARBITURIC ACID DERIVATIVE	10
BARBITAL	10
METHARBITAL	10
BUTABARBITAL	10
TALBUTAL	10
BUTETHAL	10
PRIMIDONE	10
THIOPENTAL	10
BUTALBITAL	10

THALIDOMIDE	10
HALOTHANE	10
DEXTROMETHORPHAN	10
CAFFEINE	10
YOHIMBINE	10
QUETIAPINE	10
MIANSERIN	10
ISOGUVACINE	9
ISONIPECOTIC ACID	9
MUSCIMOL	9
ANANDAMIDE	9
MIBEFRADIL	9
CYCLOTHIAZIDE	9
QUERCETIN	9
TETRAETHYLAMMONIUM	9
L-ASPARTIC ACID	9
GLYBURIDE	9
PROGESTERONE	9
LOVASTATIN	9
NICARDIPINE	9
NITRENDIPINE	9
DESIPRAMINE	9
TRIMIPRAMINE	9
ERLOTINIB	9
CLOMIPRAMINE	9
ZOTEPINE	9
TETRODOTOXIN	8
MICONAZOLE	8
VORTIOXETINE	8
CARBENOXOLONE	8
ETAZOLATE	8
ILOPERIDONE	8
PIMOZIDE	8
HYDROFLUMETHIAZIDE	8
REGORAFENIB	8
SORAFENIB	8
ISOFLURANE	8

ENFLURANE	8
ACETAZOLAMIDE	8
SUCCINIC ACID	8
XANOMELINE	8
ONDANSETRON	8
BEPRIDIL	8
CARVEDILOL	8
SIMVASTATIN	8
DOXEPIN	8
DRONEDARONE	8
PALIPERIDONE	8
ASENAPINE	8
PAROXETINE	8
BROMOCRIPTINE	8
COCAINE	7
METHIOTHEPIN	7
SERTINDOLE	7
RITANSERIN	7
METHYSERGIDE	7
EVEROLIMUS	7
KETAMINE	7
TEZAMPANEL	7
FLUNITRAZEPAM	7
VANDETANIB	7
CISPLATINUM	7
FLUMAZENIL	7
ENMD-2076	7
ARVERAPAMIL	7
ISRADIPINE	7
NISOLDIPINE	7
AMLODIPINE	7
BRINZOLAMIDE	7
CHLORTHALIDONE	7
PONATINIB	7
DESFLURANE	7
SEVOFLURANE	7
GSK2126458	7

NORTRIPTYLINE	7
PHENELZINE	7
VENLAFAXINE	7
CABERGOLINE	7
LISURIDE	7
CYT997	6
CYANOCOBALAMIN	6
LY344864	6
THIORIDAZINE	6
8-OH-DPAT	6
KETANSERIN	6
BIOTIN	6
HALOPERIDOL	6
KETAZOLAM	6
GANAXOLONE	6
[18F]FLUOROETHYLFLUMAZENIL	6
TETRAHYDRODEOXYCORTICOSTERONE	6
GABAZINE	6
BICUCULLINE	6
BRETAZENIL	6
INDOMETHACIN	6
ISONIAZID	6
VARENICLINE	6
METERGOLINE	6
TRAMADOL	6
MEPROBAMATE	6
LENVATINIB	6
DOVITINIB	6
NINTEDANIB	6
MASITINIB	6
NIMODIPINE	6
BENDROFLUMETHIAZIDE	6
TRICHLORMETHIAZIDE	6
BENZTHIAZIDE	6
METHAZOLAMIDE	6
ETHOXZOLAMIDE	6
DORZOLAMIDE	6

SORAFENIB TOSYLATE	6
RIBOFLAVIN MONOPHOSPHATE	6
BOSUTINIB	6
CARBOPLATIN	6
SODIUM BENZOATE	6
TETRAHYDROFOLIC ACID	6
AZD5363	6
PERPHENAZINE	6
PROCAINE	6
SPIRONOLACTONE	6
LITHIUM	6
MAPROTILINE	6
ROPINIROLE	6
PRAMIPEXOLE	6
TERFENADINE	6
GEFITINIB	6
ROXINDOLE	6
PERGOLIDE	6
APOMORPHINE	6
TERGURIDE	6
EPOTHILONE B	5
DL-TBOA	5
SF1126	5
DICLOFENAC	5
RETIGABINE	5
MMDA	5
NEFAZODONE	5
DONITRIPTAN	5
SPIPERONE	5
SB 216641	5
SUMATRIPTAN	5
PANOBINOSTAT	5
ANIRACETAM	5
PROPOFOL	5
ESZOPICLONE	5
ZOPICLONE	5
SURAMIN	5

DEHYDROEPIANDROSTERONE	5
CUDC-101	5
DOPAMINE	5
DB04395	5
LIDOCAINE	5
CISAPRIDE	5
NALOXONE	5
LENALIDOMIDE	5
L-CARNITINE	5
NIFLUMIC ACID	5
DULOXETINE	5
TRYPTAMINE	5
ERGOTAMINE	5
MEMANTINE	5
RGB-286638	5
BORTEZOMIB	5
PAZOPANIB	5
CINNARIZINE	5
IBUTILIDE	5
LOPERAMIDE	5
FORMIC ACID	5
DIAZOXIDE	5
METHYCLOTHIAZIDE	5
QUINETHAZONE	5
CHLOROTHIAZIDE	5
TEMSIROLIMUS	5
SELUMETINIB	5
PIMASERTIB	5
TRAMETINIB	5
METHOXYFLURANE	5
CALCIUM	5
L-ORNITHINE	5
RITONAVIR	5
BUPROPION	5
DASATINIB	5
VEMURAFENIB	5
MECAMYLAMINE	5

CYPROHEPTADINE 5
GABAPENTIN 5
TROGLITAZONE 5
MINAPRINE 5
QUINIDINE BARBITURATE 5
DILTIAZEM 5
FOLIC ACID 5
ASTEMIZOLE 5
LAMOTRIGINE 5
REPAGLINIDE 5
METHAMPHETAMINE 5
PYRUVIC ACID 5
VINBLASTINE 4
POTASSIUM CHLORIDE 4
UBIQUINONE-1 4
SAXITOXIN 4
VERATRIDINE 4
BATRACHOTOXIN 4
ETORPHINE 4
XL765 4
PI-103 4
GDC-0980 4
PKI-587 4
NOMIFENSINE 4
4-METHOXYAMPHETAMINE 4
PHENTERMINE 4
SUNITINIB 4
XE991 4
FLINDOKALNER 4
FLUPIRTINE 4
QUINIDINE 4
PSEUDOEPHEDRINE 4
TAPENTADOL 4
FENFLURAMINE 4
TEDATIOXETINE 4
MDMA 4
BIFEPRUNOX 4

PIPAMPERONE	4
EMDT	4
ELETRIPTAN	4
CYAMEMAZINE	4
TRAZODONE	4
RESMINOSTAT	4
PRACINOSTAT	4
GIVINOSTAT	4
TRICHOSTATIN A	4
SCRIPTAID	4
PCI-24781	4
VORINOSTAT	4
DACINOSTAT	4
BELINOSTAT	4
PIVANEX	4
ROMIDEPSIN	4
ZATEBRADINE	4
CILOBRADINE	4
L-AP4	4
DCG-IV	4
EGLUMEGAD	4
PERAMPANEL	4
TALAMPANEL	4
NBQX	4
AMPA	4
ATPO	4
PIRACETAM	4
FOSPROPOFOL	4
LINDANE	4
DMCM	4
ZOLPIDEM	4
BRYOSTATIN	4
GSK2636771	4
SULFASALAZINE	4
ZALEPLON	4
MODAFINIL	4
FAMOXADONE	4

UBIQUINONE-2	4
UHDBT	4
PERHEXILINE	4
CHOLIC ACID	4
2-METHOXYESTRADIOL	4
D-SERINE	4
PICROTOXININ	4
GINKGOLIDE B	4
CYTISINE	4
MESULERGINE	4
S33084	4
DAPOXETINE	4
L-PHENYLALANINE	4
FLUNARIZINE	4
NILVADIPINE	4
MAGNESIUM SULFATE	4
LEVETIRACETAM	4
ACETAZOLAMIDE SODIUM	4
DICHLORPHENAMIDE	4
L-CITRULLINE	4
IBUPROFEN	4
MK-2206	4
CITRIC ACID	4
MINOCYCLINE	4
AMPHETAMINE	4
L-CYSTEINE	4
MELATONIN	4
FLUPHENAZINE	4
HEXAMETHONIUM	4
DISOPYRAMIDE	4
PENTOXIFYLLINE	4
TUBOCURARINE	4
ERYTHROMYCIN	4
L-ALANINE	4
TOLBUTAMIDE	4
OXYMETAZOLINE	4
TRANILAST	3

IXABEPILONE	3
OLESOXIME	3
VINORELBINE	3
BUMETANIDE	3
RANOLAZINE	3
PHENYTOIN	3
ILOPROST	3
FENCLONINE	3
TETRAHYDROBIOPTERIN	3
DIHYDROMORPHINE	3
VITAMIN E	3
RO4929097	3
DEXAMETHASONE	3
RAPAMYCIN	3
ZIMELIDINE	3
AXITINIB	3
LINIFANIB	3
MOTESANIB	3
CABOZANTINIB	3
MECLOFENAMIC ACID	3
LINOPIRDINE	3
MINOXIDIL	3
AT9283	3
VX-702	3
[3H]LSD	3
AMISULPRIDE	3
RENZAPRIDE	3
LURASIDONE	3
FROVATRIPTAN	3
NARATRIPTAN	3
ZOLMITRIPTAN	3
TIOSPIRONE	3
FLUSPIRILENE	3
RIZATRIPTAN	3
QUINPIROLE	3
PINDOLOL	3
OCAPERIDONE	3

AZD8055	3
4SC-202	3
ENTINOSTAT	3
IVABRADINE	3
ENZASTAURIN	3
MPPG	3
KETOBEMIDONE	3
MILNACIPRAN	3
HOMOQUINOLINIC ACID	3
DYSIHERBAINE	3
DOMOIC ACID	3
TRANS-3-ACPBPA	3
TPMPA	3
CIS-3-ACPBPA	3
PAGOCLONE	3
ACAMPROSATE	3
CARFILZOMIB	3
BRYOSTATIN-1	3
QUINACRINE	3
PF-04691502	3
ZSTK474	3
XL147	3
GDC-0941	3
WORTMANNIN	3
NERATINIB	3
LAPATINIB	3
SIROLIMUS	3
AFATINIB	3
EGFR INHIBITOR	3
TAMOXIFEN	3
LACOSAMIDE	3
IFOSFAMIDE	3
DECITABINE	3
BIOPTERIN	3
ALFENTANIL	3
BUSPIRONE	3
FINASTERIDE	3

EPLERENONE	3
ACETAMINOPHEN	3
ATOMOXETINE	3
OXCARBAZEPINE	3
ETHINYL ESTRADIOL	3
FELBAMATE	3
MOCLOBEMIDE	3
ALBENDAZOLE	3
POMALIDOMIDE	3
NIALAMIDE	3
METHYLDOPA	3
HU-210	3
DIDS	3
ARACHIDONIC ACID	3
BILOBALIDE	3
TROPISETRON	3
PICROTIN	3
PENTOLINIUM	3
A-867744	3
QUIPAZINE	3
[3H]5-CT	3
DIHYDROERGOTAMINE	3
THIETHYLPERAZINE	3
PROMAZINE	3
PROPIOMAZINE	3
TENOCYCLIDINE	3
TC-5619	3
PURVALANOL A	3
OLOMOUCINE	3
INDIRUBIN-3'-MONOXIME	3
ALSTERPAULLONE	3
CDK1/5 INHIBITOR	3
FLAVOPIRIDOL	3
ABT-510	3
LY2835219	3
DANAZOL	3
TSU-68	3

BRIVANIB	3
SU11652	3
AMIODARONE	3
ML218	3
ABT-639	3
TTA-A2	3
AGMATINE	3
CLEVIDIPINE	3
FUROSEMIDE	3
INDISULAM	3
SULPIRIDE	3
DABRAFENIB MESYLATE	3
RAF265	3
DABRAFENIB	3
MENADIONE	3
DOXYCYCLINE	3
GALLIUM NITRATE	3
PEMETREXED	3
L-ARGININE	3
AMILORIDE	3
UREA	3
N-OMEGA-HYDROXY-L-ARGININE	3
GUANIDINE	3
RIFAMPICIN	3
IMATINIB	3
GSK2110183	3
PERIFOSINE	3
TRICIRIBINE	3
GDC-0068	3
PROMETHAZINE	3
TRIFLUOPERAZINE	3
[3H]CYTISINE	3
CHLORPROTHIXENE	3
STRYCHNINE	3
AMINOPHYLLINE	3
OXTRIPHYLLINE	3
THEOPHYLLINE	3

RIBAVIRIN	3
ISOCITRIC ACID	3
SULFINPYRAZONE	3
KETOCONAZOLE	3
COLCHICINE	3
TESMILIFENE	3
PLATINUM	3
DISULFIRAM	3
CLARITHROMYCIN	3
CLOTRIMAZOLE	3
MIFEPRISTONE	3
ALDOSTERONE	3
DOMPERIDONE	3
ACETOACETYL-COENZYME A	3
GLIPIZIDE	3
GLICLAZIDE	3
NATEGLINIDE	3
GLIMEPIRIDE	3
EPINEPHRINE	3
RAUWOLSCINE	3
